# Analysis of 5-Year-old children’s oral health service utilization and influencing factors in Guizhou Province, China (2019–2020)

**DOI:** 10.1186/s12903-023-03350-y

**Published:** 2023-09-02

**Authors:** Juanjuan Wu, Liming Chen, Min Guan, Taiming Dai, Reinhard E. Friedrich, Jiangling Sun, Wei Yang

**Affiliations:** 1 Department of Preventive Dentistry, Guiyang Stomatological Hospital, Guiyang, China; 2 Department of Oral and Maxillofacial Surgery, University Medical Center Hamburg-Eppendorf, Hamburg, Germany; 3 Department of Science and Education, Guiyang Stomatological Hospital, Guizhou, China; 4 Medical College, Guizhou University, Guiyang, Guizhou China

**Keywords:** Preschool children, Oral health service utilization, Universal health coverage

## Abstract

**Background:**

This study aimed to investigate the utilization patterns and factors related to oral health care for 5-year-old preschoolers based on Andersen’s Behavioural Model in Guizhou Province, Western China.

**Method:**

A cross-sectional study of 4,862 5-year-old preschoolers in 66 kindergartens was conducted in 2019 and 2020. A basic oral examination and a survey of parents and grandparents were conducted to gather data on oral health services. The results were analysed using chi-square tests and logistic regression analysis.

**Result:**

The utilization rate of oral health services for children in Guizhou province was 20.5%. The dmft was 4.43, and the rate of caries was 72.2%. The average cost of a dental visit was higher in rural areas and higher for girls. Logistic regression analysis revealed that dmft ≥ 6 teeth, a history of toothache, starting toothbrushing at age ≤ 3 years and limited parental knowledge were the primary factors impacting dental visits.

**Conclusion:**

Needs factors such as severe oral conditions and pain in children are the main reasons for the utilization of these services. This study underscores the urgency to actively promote the importance of oral health and expand insurance coverage for oral health services.

## Introduction

Oral diseases are among the most prevalent noncommunicable diseases, affecting approximately half of the people (3.5 billion individuals) worldwide while being mainly preventable. An estimated 2.5 billion persons have untreated tooth decay [1]. The health of deciduous teeth is intimately tied to the healthy eruption of permanent teeth, the child’s nutritional intake, and the child’s growth [2]. During the primary teeth stage, dental caries is the most prevalent oral disease [3]. Dental problems affect children’s chewing and eating, enunciation, facial development, and even their mental health as adults [4]. The dental status of a five-year-old child is critical, as this age coincides with the beginning stages of mixed dentition [5]. According to the findings of the fourth national oral health epidemiological survey conducted in China in 2017, the prevalence of early childhood caries (ECC) in 5-year-olds reached 71.9% [6].

Utilization of oral health services refers to residents’ actual use of oral health services, as well as the quantity and efficacy of oral health services provided by dental institutions to residents. It can indirectly reflect the importance that residents place on oral diseases and the level of oral health care service development [7]. It is generally accepted that children have access to oral health services in most countries. Since its inception in 1921, the School Dental Service (SDS) of New Zealand has investigated novel strategies for improvement, and children’s dental health has steadily improved since the 1980s [8]. In the United States, 94% of children who have been to the dentist for the first time continue to receive regular checkups [9]. The Fourth National Oral Health Epidemiology Survey in China provides potential predictive factors related to sourcing dental services among children, such as feeding method, initiation time of tooth brushing, and usage of fluoride toothpaste [10]. In Beijing, China, 45.5% of preschool children aged 2 to 6 years utilized oral services within 12 months [11].

Guizhou Province is located in the western region of China and is an economically undeveloped, mountainous province [12, 13]. In addition, Guizhou is a province with people of different ethnicities, including Miao, Gelao, and Buyi individuals [14]. However, Guizhou does not have as many medical resources as other southern Chinese provinces [15].

China is investigating relevant policies to lower medical costs and lighten the financial burden [16]. Reducing the financial cost of health care requires improved basic health insurance support, and it is important to include oral health services for children. It is worth noting that the utilization of oral health services for children in China is still low at present in some parts of China [10]. However, this has not been previously reported in Guizhou. Hence, to verify this claim and to explore the importance of including oral health services for children in basic public health coverage, the study aimed to conduct an epidemiological survey on the utilization of oral health services for children and related factors. For this purpose, this survey selected five-year-old children in Guizhou Province, China, and conducted a survey on them.

Several of the explanations for the significance of our findings include the following. First, the present study fills a gap in the investigation of oral health care utilization among Chinese 5-year-olds in Guizhou Province. Second, since provincial trends are not balanced across regions in China, the results of this study can be compared with those of other regions (e.g., Beijing, etc.) so that researchers can easily identify regional differences, determine their causes, and propose solutions. In addition, the local basic insurance for children does not cover dental services, and those who need dental services must bear all the costs themselves, which reduces the utilization of dental services to a certain extent and places a greater burden on preventive dental work and dentists.

In the current analysis, relevant variables were selected using the Andersen Behavioral Model as a guiding framework [17, 18]. In this investigation, we hypothesized before conducting the study that several factors might contribute to underutilization of oral health services among children in Guizhou, China, including feeding method, use of fluoride toothpaste, history of toothache and parents’ knowledge of oral health. Our study assessed these factors as independent variables.

## Method

### Study design

This survey was conducted in accordance with the STROBE guidelines [19]. This research utilized a cross-sectional design. According to local policy, a person is considered a current resident after six months of residence [20]. The inclusion criteria emphasized that participants should meet the following two requirements: residing in Guizhou Province for more than six months; being 5 years old [21]. Study participants were examined at the kindergarten school. Recruitment for this study commenced between June 2019 and November 2020. Data sources for the sample of children during recruitment included oral examinations and questionnaires (covering demographic, oral health-related behavior, evaluation of attitudes towards oral health, and oral health knowledge).

### Methods of sampling and participants

The sampling procedure used was a multistage stratified, cluster random sampling method, with the first stage using the probability proportional to size (PPS) method to select 11 districts (counties) through 9 cities in Guizhou Province; the second stage randomly selecting 6 kindergartens in each district (county); and the third stage using the whole group sampling method. Eligibility criteria for inclusion in the study were children aged 5 years, attending the sampled kindergartens, and living with parents/grandparents. The ideal sample result was 4884 people: 11 (districts) *6 (kindergartens) * 74 (people) = 4884. Twenty-two were excluded for the following reasons: inability to cooperate with the examination, absence due to illness, and parents’ refusal to sign the informed consent form. The finalized sample size was 4,862.

During the first two weeks of the agreed-upon survey, the personnel of the local health board and education administration initiated the mobilization process and distributed information and informed consent forms to the parents of the participants.

### Oral examination

Children were measured for crown caries following the WHO Basic Methods for Oral Health Surveys (5th edition) [18]. The index of decayed-missing-filled teeth (dmft) was used to measure the prevalence of primary dental caries, with data collected in accordance with World Health Organization standards [18]. According to the WHO Surveys guidance, the survey is completed in an open, quiet classroom after the examiner has cleaned the area and put on a sterilized white coat, headlamp, gloves, and set up portable dental chairs. Instruments for oral examination: portable dental chairs (Sinol, 3,052,021,070,371), sterilized plane mouth mirrors, community periodontal index (CPI) probe, rubber gloves, wash basin, and gauze are provided. 

#### Questionnaire

The variables included in the questionnaire were derived from the guidance provided by China’s Fourth Oral Epidemiological Survey and the Oral Health Surveys Basic Methods (5th edition, 2013) published by the World Health Organization [10, 22]. The outcome of the study pertains to children aged 5 who have utilized oral health services at any point in their lives. This includes various aspects such as community oral health services, seeking medical attention, and participation in oral health promotion activities [10]. The selection of items and measures was conducted through extensive discussions among Chinese public health experts, medical statistics experts, and dentistry experts. This collaborative process ensured the relevance and appropriateness of the constructs being assessed in the study. The relevant variables have also been validated in other regions, such as Beijing, China [11]. Face-to-face interviews with the children’s parents/grandparents were used to carry out the questionnaire, which asked them questions about the children’s demographic variables (such as sex, birth year, ethnicity, type of household, respondent, birth weight, feeding method) and oral health-related behaviour variables (eating habits and consumption frequency of sweets, brushing frequency, toothpaste use, dental visit experience, etc.). Evaluation of attitudes regarding oral health (if oral health is vital to one’s life, whether healthy or unhealthy parental teeth would affect those of their children, whether routine oral exams are needed, and whether dental disorders need to be prevented by oneself first), oral health knowledge Q&A (including whether it is normal for gums to bleed due to brushing, whether bacteria can cause gum inflammation, the role of brushing in preventing gum bleeding, whether bacteria can cause tooth decay, whether eating sugar can cause tooth decay, whether bad deciduous teeth need to be treated, whether pit and fissure sealing can prevent tooth decay in children, and whether the use of fluoride toothpaste can prevent tooth decay), parents educational experience, annual family income, etc., were assessed. The oral health knowledge survey consisted of eight questions, with 1 point awarded for a correct response and 0 points deducted for an incorrect response. Received 5 or more points, denoting performance ≥ 60%. The oral health attitude questionnaire consisted of four questions, all of which supported what was taken as a good oral health attitude.

#### Conceptual model

According to Andersen Model [17], factors that predispose, enabling, and create the need for seeking health care are the basis for this study.

Predisposing factors were categorized as clinical measures, eating/feeding factors, cleaning-related behaviours, and parental oral health knowledge and attitudes among these. Parents’ education and annual family income are categorized as enabling factors. The remaining need factors include dmft and dental pain experiences.

#### Quality control

The 6 examiners were licenced dentists who had received standardized training and calibration from dentists with 20 years of experience prior to the study, and 5% of respondents were chosen for calibration during the on-site survey. Kappa coefficients were used to assess the consistency between different examiners (inter-examiner reliability) and within the same examiner at different time points (intra-examiner reliability) for the dmft index, following the guidelines outlined in the Oral Health Surveys Basic Methods [22]. Kappa values were evaluated using the categorization system developed by Landis and Koch [23]. The kappa value of 0.61–0.80 indicates substantial agreement, while a kappa value of 0.81-1.00 represents almost perfect agreement. Both inter-examiner reliability and intra-examiner reliability achieved substantial agreement and demonstrated high reliability. The Kappa coefficient for inter-examiner reliability was 0.80, while the Kappa coefficients for intra-examiner reliability ranged from 0.80 to 1.00.

### Statistical analysis

Two trained professionals conducted the data collection, including the oral exam and questionnaire components. The data were double-entered into a self-built oral prevention data platform. In addition, the results were statistically analysed using SPSS 24.0 software. To account for the complex sampling design, weighted statistical methods were employed to adjust for potential bias introduced by the multistage stratified, cluster random sampling method. Comparisons between urban and rural groups, as well as male and female groups, were conducted using chi-square tests with nonparametric tests, and the relevant influencing factors were analysed using logistic regression with a significance level of α = 0.05.

## Result

### Basic data regarding survey responders

A total of 4862 children aged 5 years were surveyed in Guizhou province, including 2436 males (50.1%) and 2426 females (49.9%) who had the following characteristics: 2202 had urban (45.3%) and 2660 had rural (54.7%) residences; the main ethnic distribution was 2899 Han (59.6%), 916 Miao (18.8%), 374 Buyi (7.7%), Tujia 226 (4.6%), Dong 129 (2.7%), and other 318 (6.6%); caries filling ratio was 1.7%, i.e., 98.3% of dental cavities remained untreated. The caries rate for the group that brushes their teeth before the age of three was 73.2%, while after the age of three, it was 71.7%. Dmft was 4.43, of which decayed teeth (dt) comprised 98.8%, filled teeth (ft) comprised 1.1%, and missing teeth (mt) comprised 0.1% in the survey.

### Caries prevalence among 5-year-old children in Guizhou Province

The caries rate of primary teeth in the 5-year-old group was 72.2%, with a statistically significant difference between participants living in rural (79.8%) and urban (67.6%) areas (*χ*^2^ = 53.162, *p* < 0.001) and a statistically significant difference in dmft between participants residing in rural (5.19) and urban (3.98) areas (t = 54.441, *p* < 0.001).

**Fig. 1:**
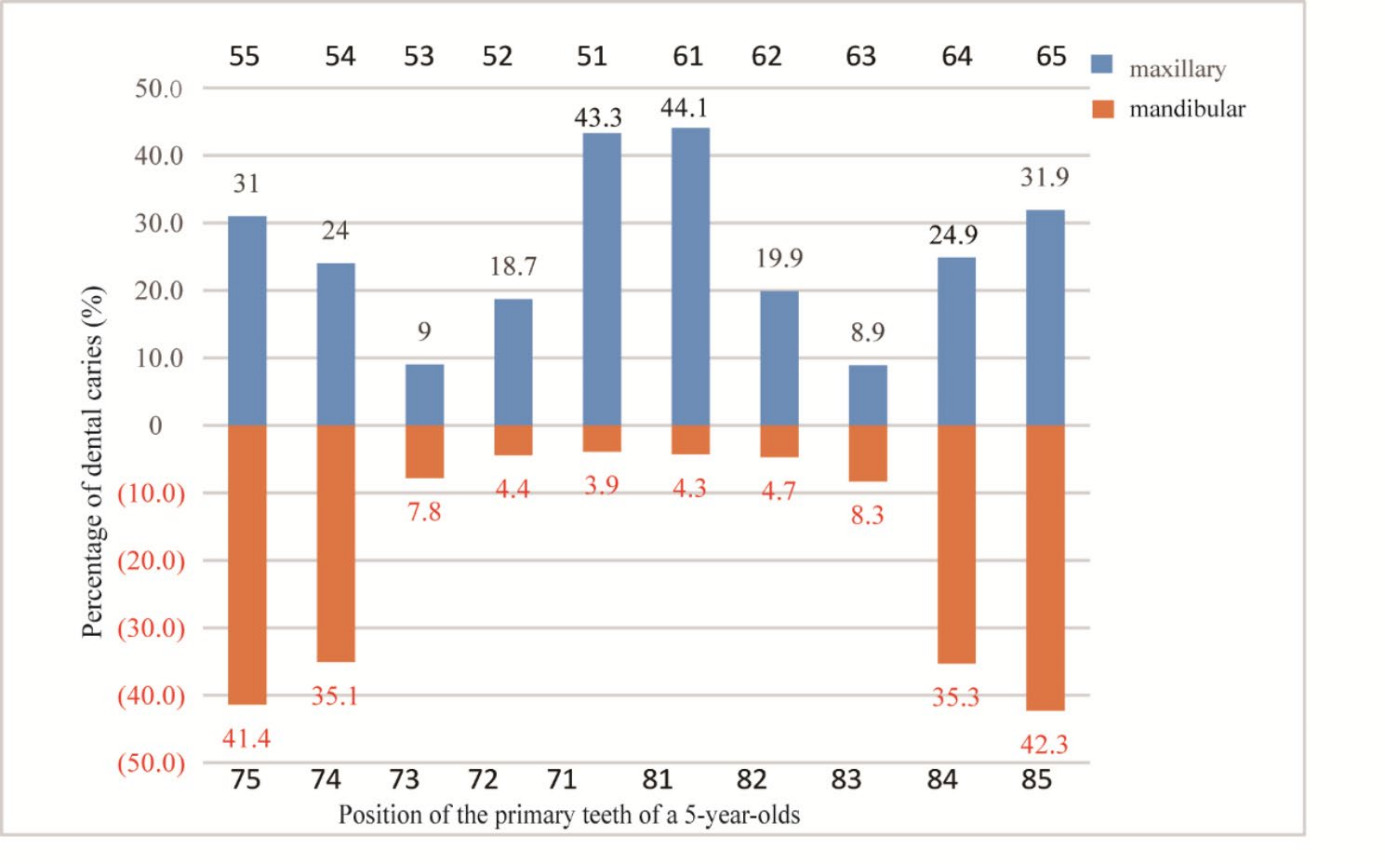
Caries prevalence among 5-year-old children in Guizhou Province from 2019–2020

In the 5-year-old group, the teeth with the highest caries prevalence were the primary maxillary incisors and the mandibular second primary molars (Fig. 1). Using the Fédération Dentaire Internationale Two-Digit System, the position of the primary teeth was recorded [24].

### Oral health service utilization among 5-year-old children in Guizhou Province

At age 5, the rate of dental visits for children in Guizhou province was 20.5%. Experience rates in Guizhou province were significantly higher in rural areas (21.4%) than in urban areas (20%) and among girls (21.5%) than among boys (19.6%) (Table 1).

**Table 1:** Distribution of oral health service utilization and last dental visit among 5-year-olds in Guizhou Province

	Survey Number of people	Experienced in oral health service utilizatio				Time of last utilization of oral health services (%)		
		Number of people	Utiliation rate of oral health services (%) Weighted	^*2*^	*p*	< 6 months	6–12 months	> 12months
Urban	2202	533	20.0			42.0	26.4	31.6
Rural	2660	591	21.4	11.801	< 0.001	45.2	22.8	32.0
Male	2436	545	19.6			44.4	25.3	30.4
Female	2426	579	21.5	21.833	< 0.001	42.2	24.8	33.1
Total	4862	1124	20.5			43.2	25.0	31.8

### Oral health service utilization among children aged 5 years in 12 months in Guizhou Province

In Guizhou province, 14.0% of 5-year-olds received oral medical care within 12 months (Table 2), with no statistically significant variations between rural and urban areas or between males and females (*χ*^*2*^ = 0.542, *p =* 0.462; *χ*^*2*^ = 2.863, *p =* 0.094). The average cost of a dental visit over 12 months was 633.19 Chinese Yuan and was higher in rural than in urban areas and higher in girls than in boys (*Z*=-3.822, *p <* 0.001; *Z*=-2.653, *p =* 0.008).

**Table 2:** Principal reasons for 12-month oral health service utilization, average cost, and last dental visit among 5-year-olds in Guizhou Province

	Survey Number of people	oral health service utilization within 12 months		*χ* ^*2*^	*p*	Average cost (¥) of utilizing oral health services in the past twelve months		*Z*	*p*	Reason for oral health service utilization within 12 months (%)			
		Number of people	Rate (%) weighted			X‾	*S*			Consultation check	Prevention	Treatment	Do not know
Urban	2202	388	13.7	0.542	0.462	579.59	1.036,69	-3.822	< 0.001	40.6	13.0	45.9	0.6
Rural	2660	413	14.6			724.93	1.537,34			37.6	18.6	41.6	2.2
Male	2436	388	13.7	2.863	0.094	517.53	1.041,37	-2.653	0.008	39.1	15.4	43.8	1.7
Female	2426	413	14.4			759.43	1.427,44			39.7	15.0	44.6	0.7
Total	4862	801	14.0			633.19	1.246,68			39.4	15.2	44.2	1.2

### Factors influencing the usage of paediatric oral health services

In the univariate analysis of children attending versus not attending paediatric oral health services (Table 3), factors such as oral health status and sociodemographic characteristics of survey respondents were included. The data show that the decision to attend a dental visit for a 5-year-old child in Guizhou province is related to several factors (the number of caries teeth, eating before sleep, whether they brush their teeth, age of starting to brush their teeth, parents recently helping their children brush their teeth, use of fluoride toothpaste, history of toothache within the last 12 months, parents’ knowledge of oral health, parents’ education, and annual family income). There were statistically significant differences *(p* < 0.05).

**Table 3:** presents the outcomes of a univariate study of the current status of oral health service utilization among 5-year-olds in Guizhou Province

	Classification	Total number of cases (composition ratio)	Cases	Attendance rate	*χ* ^*2*^	*p*
dmft	≥ 6	2036 (41.9%)	716	35.2%	271.415	< 0.001
	< 6	2826 (58.1%)	408	14.4%		
Birth Weight (kg)	2.5 ≥ BW ≥ 4	3865 (79.5%)	942	24.4%	0.000	0.999
	BW < 2.5 or over 4 kg	391 (8.0%)	82	21.0%		
	Unknown	606 (12.5%)	100	16.5%		
Feeding Method	Breastfeeding	2228 (45.8%)	511	22.9%	0.011	0.997
	Artificial feeding	601 (12.4%)	135	22.5%		
	Mixed feeding	2033 (41.8%)	478	23.5%		
Dessert Consumption (number of times)	≥ 1	851 (17.5%)	231	27.1%	3.669	0.145
	< 1	4011 (82.5%)	893	22.3%		
Sugary Drinks (times/day)	≥ 1	408 (8.4%)	118	28.9%	0.007	0.951
	< 1	4454 (91.6%)	1006	22.6%		
Sugar-sweetened Milk (times/day)	≥ 1	807 (16.6%)	220	27.3%	0.007	0.951
	< 1	4055 (83.4%)	904	22.3%		
Eating before Sleep	Yes	2833 (58.3%)	717	25.3%	9.606	0.024
	No	2029 (41.7%)	407	20.1%		
Brushing Teeth Every Day	Yes	3872 (79.6%)	966	24.9%	43.549	< 0.001
	No	990 (20.4%)	158	16.0%		
Age of Starting to Brush Teeth (years)	< 3	1264 (26.0%)	389	30.8%	34.696	< 0.001
	≥ 3	2848 (58.6%)	613	21.5%		
	Unknown	750 (15.4%)	122	16.3%		
Parents recently helped their children brush teeth	Yes	1615 (33.2%)	494	30.6%	57.996	< 0.001
	No	2598 (53.4%)	527	20.3%		
	Unknown	649 (13.3%)	103	15.9%		
Use of fluoride toothpaste	Yes	476 (9.8%)	146	30.7%	14.908	0.005
	No	4386 (90.2%)	978	22.3%		
History of toothache within last 12 months	Yes	1441 (29.6%)	410	46.8%	581.743	< 0.001
	No	3165 (65.1%)	675	13.0%		
	unknown	256 (5.3%)	39	15.2%		
Oral health attitude of parents	Good	766 (15.8%)	195	25.5%	1.444	0.361
	Not so good	4096 (84.2%)	929	22.7%		
Parents’ knowledge of oral health (%)	≥ 60%	2666 (54.8%)	706	14.5%	38.575	< 0.001
	< 60%	2196 (45.2%)	418	19.0%		
Parents’ education	Middle school and below	3349 (68.9%)	663	19.8%	55.452	< 0.001
	High school	1156 (23.8%)	339	29.3%		
	Bachelor’s degree or above	357 (7.3%)	122	34.2%		
Annual family income (¥ Chinese Yuan)	≥ 20,000	622 (12.8%)	170	27.3%	11.563	0.011
	< 20,000	1944 (40.0%)	434	22.3%		
	unknown	2296 (47.2%)	520	22.6%		

### Logistic regression investigation of characteristics associated with children’s use of oral health services

By univariate analysis, several factors were identified to be included in the multifactorial logistic regression analysis of dental visits. The results revealed that dmft ≥ 6, a history of toothache within the last 12 months, beginning toothbrushing at less than 3 years of age, and limited parental knowledge were the primary factors impacting the use of oral health services **(**Table 4**)**.

**Table 4:** Logistic regression analysis of factors associated with the use of oral health services for children

		*β*	wald*X*^*2*^	*P*	OR	95% CI
From urban or rural	Urban	0.140	0.890	0.346	1.150	0.86–1.539
	Rural					
Sex	Male	-0.098	0.409	0.523	0.907	0.673–1.223
	Female					
dmft	dmft ≥ 6	0.433	7.534	0.006	1.543	1.132–2.102
	dmft < 6					
Eating before sleep	Yes	-0.134	0.716	0.397	0.874	0.641–1.193
	No					
Brush teeth everyday	Yes	-0.108	0.145	0.703	1.114	0.638–1.946
	No					
Age of starting to brush teeth (years)	< 3	0.394	6.120	0.013	1.483	1.085–2.026
	≥ 3					
Parents recently helped their children brush teeth	Yes	0.229	2.151	0.143	1.257	0.926–1.708
	No					
Use of fluoride toothpaste	Yes	-0.203	0.732	0.392	0.816	0.512–1.301
	No					
Parents’ knowledge of oral health (%)	≥ 60%	0.335	4.280	0.039	1.398	1.018–1.920
	<60%					
History of toothache within last 12 months	Yes	1.605	100.690	< 0.001	4.978	3.638–6.812
	No					
Parent’s education	Middle School and below	-0.198	0.297	0.441	0.820	0.495–1.358
	High School	-0.159		0.559	0.853	0.501–1.453
	Bachelor’s degree or above					
Annual family income (¥)	≥ 20,000	0.063	0.128	0.720	1.066	0.752–1.509
	< 20,000					

## Discussion

Oral health, especially in children, is currently a medical challenge. This is due to the consumption of a variety of sugary foods and poor oral habits, which can have a negative impact on general and mental health [1]. The oral health of a population may be worsen by low visit rates and financial burden. Our 2019–2020 study examined oral health service utilization and associated factors among 5-year-olds in Guizhou Province.

The results of this study showed that the utilization of oral health services for children in Guizhou province was 20.5% (including dmft 4.43, caries rate 72.2%, and untreated caries 98.3%). This could be attributed to the fact that the average cost of a dental visit was higher in rural areas and for girls. Moreover, logistic regression analysis showed that dental caries ≥ 6 teeth, a history of toothache, starting toothbrushing at less than 3 years of age, and limited parental knowledge were the most important factors impacting dental visits.

A ten-year examination of Brazilian preschoolers revealed that the average dmft had decreased from 1.88 to 2006 to 0.99 in 2016 and that up to 78% of children had reached caries-free status [25]. In Germany, the prevalence and incidence of caries among children aged 5 were 26.2% and 0.9 ± 2.0 dmft, respectively, in 2015 [26]. In this study, from 2019 to 2020, 5-year-olds in Guizhou Province in China had a dmft of 4.43 and a caries rate of 72.2%. However, the study conducted by this group from 2015 to 2016 revealed that the prevalence of dental caries in children aged 3–5 in Guizhou was 63.1%, and the mean dmft of ECC in children aged 3–5 in Guizhou province was 3.32 [27]. From these numbers, it is clear that the dental caries of 5-year-olds in Guizhou are much higher than those in other nations, even greater than the survey results provided from four years ago, and that action needs to be taken by the local medical administration and dentists.

According to the Chinese Fourth National Oral Epidemiological Survey, the overall dental visit rate for children aged 5 in China is 25.4%, and the attendance rate in the last 12 months is 19.2% [10]. In this study, at age 5, the rate of dental appointments for children in Guizhou province was 20.5%, and the attendance rate in the last 12 months was 14.0%. Guizhou Province, unlike other developed provinces in China (e.g., 21.5% for children in Guangdong Province and 15.63% for children in Zhejiang Province), has a relatively low incidence of 5-year-olds seeing the dentist in the previous 12 months [28, 29].

Caries is the most common form of oral illness in children and the leading cause of dental visits [30]. This survey showed that 98.3% of open cavities were not filled. In addition, the cost of a dental appointment for a 5-year-old child in Guizhou province was 633.19 Chinese Yuan, almost entirely self-pay, which is more than 413.65 Chinese Yuan nationally [10]. The aforementioned demonstrates that 5-year-olds in Guizhou Province have low utilization of oral health services and a significant economic burden of oral diseases.

At the age of 5, dental caries rates are higher among rural children than among urban children, so rural children ought to have a higher attendance rate. However, the difference between rural and urban children is not statistically significant, likely due to a lack of oral health care services in rural areas, consequently, some rural children might skip a consultation or treatment. Some children’s parents choose to travel to urban areas for oral care services. Considering travel time and road and hotel expenses, the majority of rural children will have a single treatment for multiple teeth or complete the entire treatment at once, resulting in a higher cost. We assume that this is the reason that there is no difference in attendance between rural and urban areas but that there is a difference in cost, although further investigation is needed.

In this study, a multifactor logistic regression study revealed that dental caries ≥ 6, a history of toothache for the past 12 months, age of starting to brush teeth before 3 years, and inadequate parents’ knowledge of oral health were the most influential factors in the utilization of oral health services. The high dental visit rate of children with dental caries ≥ 6 cavities and a history of toothache within the last 12 months suggests that the utilization of dental services for 5-year-olds in Guizhou Province continues to be demand-driven. In addition, children at this age are in the early phases of mixed dentition, and the growth of permanent teeth, such as the first molars, can be associated with dental pain [31], hence increasing the need for dental consultations.

A 5-year-old youngster with more than six teeth with cavities is deemed to have severe underage caries [32]. It takes a long time for caries to reach a painful stage, and this study indicated that many parents would only take their children for treatment if caries had reached a severe stage, indicating that the actual need for dental care is significantly higher than the demand for dental visits [11]. There is one possible explanation for the higher dental visits of children’s parents who begin brushing their teeth before the age of three. This is similar to this group’s previous study in that children who brush more frequently have higher caries rates [27]. The caries rate was higher among children who began brushing their teeth before the age of three (73.2%) than among children who began brushing their teeth after three (71.7%). It can be inferred that some of the children who started brushing their teeth under the age of three may have already developed caries before their parents chose to brush their teeth or seek medical attention. Parents are primarily responsible for the oral health of their newborns and early children, and a lack of parental health knowledge is associated with a deterioration in the oral health status of children and an increase in their likelihood of seeking medical assistance [33].

Ultimately, comparing the current results of oral status of 5-year-olds and policies regarding other countries or regions [34–36], passive attendance is a key characteristic of 5-year-old children’s utilization of oral health services in Guizhou province and that a shift from a passive to an active attitude is a direction in which to work. According to the draft Global Oral Health Action Plan (2023–2030), by 2030, 75% of the global population will be covered by essential oral health care services to ensure progress towards Universal Health Coverage (UHC) for oral health [37]. UHC indicates that all individuals and communities have access to essential, high-quality health services that meet their needs and that they can use without experiencing financial hardship [37]. In recent years, the Lancet has published article proposals for incorporating basic oral health services and necessary oral care packages into universal health programs [38]. Although children in Guizhou, China, are largely covered by basic health insurance, children’s oral health care is not included. The fact that children’s oral health treatments are completely self-funded by their parents may contribute to the low dental visit rate. Children of this age group in the region require a high level of dental care to reduce dental caries.

Three suggestions can be made to improve the utilization of oral services for preschool children in Guizhou province. First, oral health education for pregnant women and young parents should be enhanced so that they are concerned about dental health and recognize the need for regular checkups. Second, the system of oral health consultation services based in kindergartens needs be expanded so that oral health issues can be diagnosed and treated on campus. Third, it is proposed that the government cover the expense of dental services for children in basic health insurance to reduce the financial burden of oral problems on families.

Limitations: this study harbors several limitations that should be acknowledged. First and foremost, a considerable limitation is the use of cross-sectional study design, which precludes the ability to establish causality between the associated factors and utilization of oral health services. Moreover, the study was confined to 5-year-old preschoolers in Guizhou Province. This geographical and age limitation may prevent the generalizability of the findings to other regions or age groups. In addition, reliance on self-reported data from parents and grandparents, could introduce recall bias, influencing the accuracy of the data. Additionally, the reasons why 5-year-old females had a higher expenditure on dental visits in the past 12 months weren’t fully explored, warranting further research.

## Conclusion

The utilization of oral health services among 5-year-old children in Guizhou Province is significantly associated with the level of importance attributed by the parents. Need factors such as severe oral conditions and pain in children are the main reasons for the utilization of these services. This study underscores the urgency to actively promote the importance of oral health and expand the insurance coverage for oral health services.

## Data Availability

The dataset used and/or analyzed during the current investigation is available upon reasonable request from the corresponding author.
